# Comment on “CT findings in surgically treated focal pancreatic disease – a retrospective study”

**DOI:** 10.1097/MS9.0000000000004560

**Published:** 2025-12-16

**Authors:** Muhammad Abdur Rahman Aizaz, Sakan Binte Imran

**Affiliations:** a Department of Medicine, Jinnah Sindh Medical University, Karachi, Pakistan; b Department of Medicine, Sir Salimullah Medical College, Dhaka, Bangladesh

We read with great interest the article by Munk-Madsen *et al*. The authors have investigated an important and often confusing clinical challenge – differentiating autoimmune and chronic pancreatitis (CP) from pancreatic ductal adenocarcinoma (PDAC) using CT imaging^[1]^. Their attempt to improve imaging characteristics in surgically treated patients adds a meaningful contribution to pancreatic diagnostics. That said, we would like to offer a few reflections that might be useful for future research on this topic. This editorial aligns with the TITAN 2025 guidelines^[2]^.

First, the study does not distinguish between type 1 and type 2 autoimmune pancreatitis (AIP). These subtypes differ in their underlying mechanisms, age distribution, and IgG4 profiles; analyzing them together may obscure important subtype-specific imaging patterns and clinical insights^[3]^. By analyzing them collectively, the study may have missed minor but meaningful differences in imaging features and clinical behavior. In everyday clinical practice, identifying the correct subtype is crucial for guiding diagnosis and treatment. Further studies should include subtype categorization.

The study effectively draws attention to cases where AIP or CP were mistaken for PDAC, which is an important clinical concern. However, it does not tell us what became of those patients whether they faced complications, long-term effects, or changes in their quality of life after being misdiagnosed^[4]^. Understanding these consequences would make the findings far more meaningful and emphasize why improving diagnostic accuracy truly matters for patients. Further studies should include longitudinal follow-up of misdiagnosed cases.

The study acknowledges the importance of IgG4 and CA19-9 in differentiating AIP from PDAC; however, these markers were not consistently tested across cases^[5]^. Moreover, their diagnostic performance such as sensitivity and specificity was not examined. Including these markers in a combined diagnostic model could have added significant clinical value. Without this integration, the study’s diagnostic approach is incomplete and less applicable to real-world practice. Further studies should effectively utilize biochemical markers.

In conclusion, this study provides valuable insight into the ongoing challenge of distinguishing benign pancreatic diseases from cancer. Future studies that separate AIP subtypes, follow patients over time, and make better use of biochemical markers would add real depth to our understanding and make the findings more relevant to future clinical research and diagnostic practice.
